# New Drugs for Neglected Diseases: From Pipeline to Patients

**DOI:** 10.1371/journal.pmed.0010006

**Published:** 2004-10-19

**Authors:** Bernard Pécoul

## Abstract

The Drugs for Neglected Diseases Initiative is a new, public- sector organization dedicated to drug discovery

In wealthy countries, state-funded research has yielded breakthroughs in molecular biology, chemistry, and engineering. These advances have been taken up by the pharmaceutical industry and applied to drug development for a growing range of illnesses and conditions. As a result, patients have access to new drugs that are better tolerated, more specific, and more effective than old ones.

In poor countries, however, millions of people have yet to experience the benefits wrought by science. The deadly infectious diseases that plague them, such as sleeping sickness, Chagas disease, and visceral leishmaniasis, fail to arouse the interest of drug developers.

The Drugs for Neglected Diseases Initiative (DNDi) is a new, not-for-profit organisation set up to correct this fatal imbalance by developing new drugs for these forgotten patients.

## Dropped off the Radar Screen

Most of the drugs still used to treat ‘neglected diseases’ were developed in colonial times. These are often expensive, difficult to administer, and hard to tolerate; several of them are also becoming ineffective because of increasing parasite resistance. Very few new alternatives have been developed in the past decades: between 1975 and 1999, 1,393 new drugs were made available to the public, but only 16 of these were meant for neglected diseases [1].

**Figure 1:**
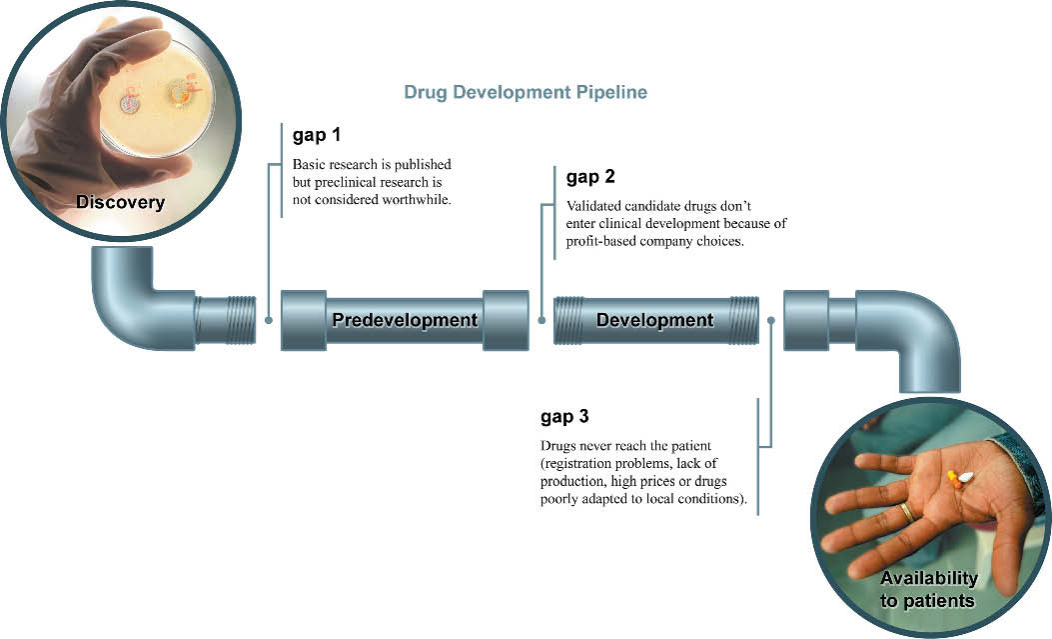
The Drug Development Pipeline Because of the gaps in the development pipeline, potential new drugs for neglected diseases often stay stuck at an early stage of development. (Photos: World Health Organization/P.Virot and World Health Organization/Eric Miller)

What makes the lack of drugs more difficult to accept is that scientists know an enormous amount about kinetoplastids, the organisms responsible for sleeping sickness, Chagas disease, and leishmaniasis [2]. The wealth of knowledge generated in this field could easily be used for drug development if the treatment of neglected diseases were perceived as financially attractive. But populations affected by neglected diseases have no purchasing power, so there is no financial incentive for drug companies to develop the drugs. The basic mechanics of the market-driven system are failing to help these populations. So most scientific research stops at the publication stage or falls through the gaps at different stages of the drug development pipeline (Figure 1) [3].

## Whose Role Is It, Anyway?

It is dangerous to oversimplify the causes of this situation. What share of responsibility for the world's health is borne by the pharmaceutical industry, which has the know-how and the resources for innovation? Aren't international organisations also partly responsible? After all, they are the ones who allocate major funding for health programmes and encourage research programmes. And what about public research institutions in rich countries that generate the knowledge used by industry? Governments have the power to influence their research priorities and drug development decisions, either through funding or direct involvement. Unfortunately drugs for neglected diseases are low priority for governments [4]. They tend to prioritise research with potential commercial applications instead.

## Responses to the Crisis

All is not doom and gloom. In the past few years, there has been some movement on research and development (R&D) for neglected diseases. Despite its broad mandate and limited resources, the Special Programme for Research and Training in Tropical Diseases (TDR)—established and funded by the World Health Organization, the World Bank, and the United Nations Development Programme—can be credited with several important successes in the fight against malaria and leishmaniasis. The Medicines for Malaria Venture and the Global Alliance for TB Drug Development were set up as public–private partnerships to tackle malaria and tuberculosis. These partnerships were made possible by the fact that malaria and tuberculosis are global diseases, affecting patients in the North and South, so there was enough of a market to persuade industry to develop new drugs for these diseases.

A different solution, however, was needed for diseases that are limited to tropical countries, are of no military or strategic interest to wealthy countries, and are not supported by markets or patients' organisations capable of attracting the attention of politicians. This is the kind of solution put forward by the DNDi.

## A Collaborative Not-for-Profit

DNDi is a not-for-profit organisation designed to mobilise resources for R&D of new drugs for neglected diseases.

Many people and organisations around the world share an ambition to redress the lack of new treatments for neglected diseases, and bring the benefits of science to forgotten patients. Several of them came together to create DNDi: one humanitarian organisation—Médecins Sans Frontières; five research institutions—the Oswaldo Cruz Foundation from Brazil, the Indian Council for Medical Research, the Kenya Medical Research Institute, the Ministry of Health Malaysia, and the Pasteur Institute from France; and the TDR (Box 1).

**Box 1. From Pipeline to Patients—Some Key Organizations**

**DNDi:** http://www.dndi.org

**TDR:** http://www.who.int/tdr

**Medicines for Malaria Venture:** http://www.mmv.org

**Global Alliance for TB Drug Development:** http://www.tballiance.org

**Oswaldo Cruz Foundation:** http://www.fiocruz.br

**Indian Council for Medical Research:** http://icmr.nic.in/home.htm

**Kenya Medical Research Institute:** http://www.kemri.org

**Ministry of Health Malaysia:** http://dph.gov.my/

**Pasteur Institute:** http://www.pasteur.fr/externe

**Médecins Sans Frontières:** http://www.msf.org

The initiative is a virtual organisation with a growing network of academic and R&D expertise at its disposal. The different players involved in DNDi are bringing their knowhow in parasitology and clinical trials, their experience treating neglected patients, and their drug manufacturing capacity. They are pooling these resources to move drugs stuck in the pipeline all the way to the patients themselves. Pharmaceutical companies have a particularly important role to play: they possess vast repositories of molecules, the means to move from development to industrial production, and highly specialised teams of researchers. Their contribution will be crucial to the success of DNDi.

## Matching Needs and Opportunities

DNDi is a needs-driven initiative—in other words, the needs of patients suffering from neglected diseases are paramount in its search for new drugs to treat them. The organisations that make up DNDi have firsthand knowledge of these needs because they work with patients in disease-endemic countries throughout the developing world. The initiative is taking this knowledge of patient needs, matching it with opportunities in R&D, and pushing the most relevant projects through the pipeline. Ultimately, neglected patients will have access to drugs targeting their specific diseases, drugs that were designed with them specifically in mind—such as short-course, low-toxicity treatments that don't require hospitalisation, or tablets to swallow rather than injections.

To identify opportunities in R&D that are both relevant to patient needs and that meet required criteria of scientific merit, DNDi is sending out calls for letters of interest to the scientific community via advertisements in journals and posted on the DNDi website (http://www.dndi.org). These have already pinpointed several promising projects. DNDi is also proactively contacting scientists working on infectious diseases, and surveying published literature for research of interest.

## In the Pipeline

**Figure 2:**
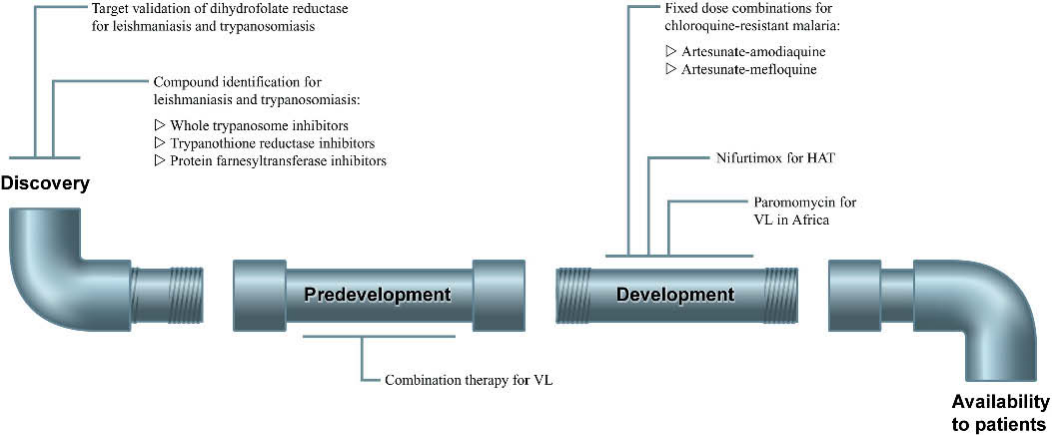
DNDi Projects DNDi's project portfolio contains nine projects spread out across the drug development pipeline for the treatment of leishmaniasis, sleeping sickness, Chagas disease, and malaria. HAT, human African trypanosomiasis (sleeping sickness); VL, visceral leishmaniasis.

DNDi's project portfolio currently holds nine projects at different stages of development to address identified needs for the treatment of visceral leishmaniasis, sleeping sickness (Box 2), Chagas disease, and malaria (Figure 2). At discovery stage, DNDi is working on validating the kinetoplastid enzyme dihydrofolate reductase as a potential target for leishmaniasis, trypanosomiasis, and Chagas disease, and on identifying inhibitors of the kinetoplastid enzymes trypanothione reductase and protein farnesyltransferase. It is also conducting high throughput screening on whole cell trypanosomes to discover novel lead compounds.

**Box 2. New Drugs for Sleeping Sickness**

Only a few drugs exist to treat sleeping sickness, and they are toxic or difficult to administer. Melarsoprol kills one in 20 patients. Eflornithine requires four daily infusions over 14 days. Given these limited options, DNDi is focusing on identifying new compounds that can cross the blood–brain barrier to treat second stage sleeping sickness.

DNDi is using high throughput screening on whole cell trypanosomes to discover novel lead compounds, and is working to identify and optimise inhibitors of the enzyme protein farnesyltransferase. The initiative is working on validating the kinetoplastid enzyme dihydrofolate reductase as a drug target. Identifying trypanothione inhibitors is also relevant to other trypanosome parasites. These are long term projects.

Nifurtimox, a drug for Chagas disease, has been used to treat sleeping sickness since the 1970s in some isolated places. It has never been extended to more people because no one has studied its safety or effectiveness. DNDi will assess its short-term usefulness by conducting clinical trials on a treatment combination of eflornithine and nifurtimox. DNDi will continue to explore other short- and medium-term projects.

The R&D of new drugs is time-consuming and expensive if the process starts at the early discovery stage, because of the associated risk of project attrition along the way. DNDi is therefore investing resources in several pre-development and development projects as well. These include developing fixed dose combinations of artesunate/amodiaquine and artesunate/mefloquine for use against chloroquine-resistant malaria in Africa and Asia, respectively; pushing for registration of paromomycin for use against visceral leishmaniasis in Africa; assessing combinations of existing drugs for visceral leishmaniasis; and evaluating the usefulness of nifurtimox in combination with eflornithine in the treatment of sleeping sickness.

## Advocacy for Change

Governments can—some might say should—influence drug development choices. DNDi strongly believes that governments in both developed and developing countries should take an active interest in the R&D of new drugs for neglected diseases. In parallel to its own drug development activities, DNDi is working to raise awareness of the neglected disease crisis among key policy- and decision-makers, for instance the European Commission and the National Institutes for Health in the United States.

## Conclusion

In the poorer countries in the world, over 350 million people are at risk from neglected diseases. Currently available treatments are inadequate or nonexistent, and new solutions are urgently needed. DNDi is working to ensure that the advances of science that have brought health and comfort to wealthy nations also benefit these neglected populations.

## Abbreviations

DNDi: Drugs for Neglected Diseases Initiative
R&D: research and development
TDR: UNICEF/UNDP/World Bank/World Health Organization Special Programme for Research and Training in Tropical Diseases
WHO: World Health Organization
